# Supplementary material for: EGFR Exon 20 Insertion in Metastatic Non-Small-Cell Lung Cancer: Survival and Clinical Efficacy of EGFR Tyrosine-Kinase Inhibitor and Chemotherapy
Source: Cancers (Basel). 2021 Oct 13;13(20):5132. doi: 10.3390/cancers13205132 (PMC8534282; doi:10.3390/cancers13205132)
Supplement: Supplementary file 1 [file cancers-13-05132-s001.zip › cancers-1238459-supplementary.pdf]

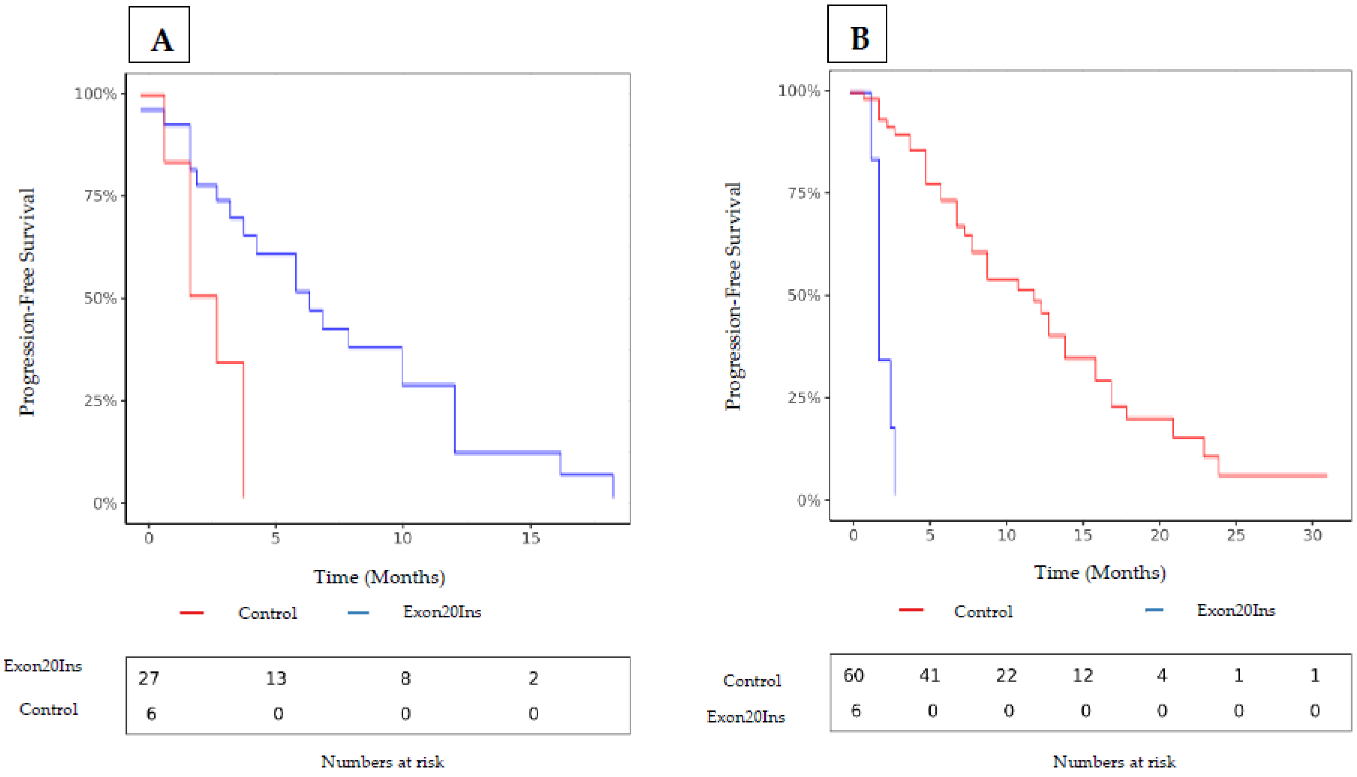

Supplementary Figure S1. Kaplan-Meier estimation of Progression-free survival (PFS) measured from inclusion in patients with Exon 20 insertion (blue) and control (red) receiving first-line chemotherapy (Panel A) and receiving first-line TKIs (Panel B).

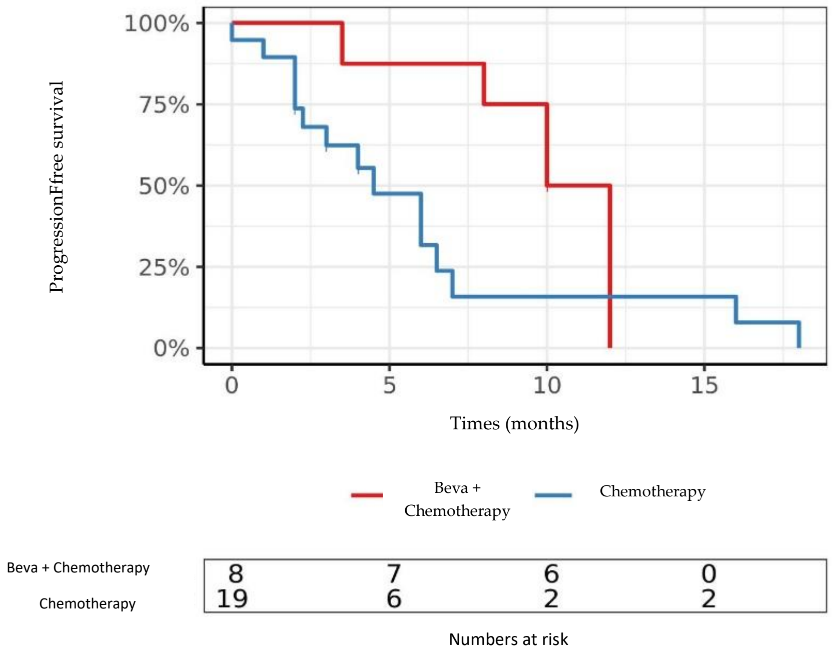

Supplementary Figure S2. Kaplan-Meier estimation of Progression-free survival (PFS) measured from inclusion in patients with Exon 20 insertion receiving platin-based chemotherapy alone (blue, n=19) and platin-based chemotherapy + bevacizumab (red, n= 8): PFS= 4.5 vs 11 months, respectively (p=0.1).
